# Expression of thymidylate synthase in human cells is an early G_1_ event regulated by CDK4 and p16INK4A but not E2F

**DOI:** 10.1038/sj.bjc.6604020

**Published:** 2007-10-09

**Authors:** B G Le François, J A Maroun, H C Birnboim

**Affiliations:** 1 Department of Biochemistry Microbiology and Immunology, University of Ottawa, Ottawa, Ontario, Canada; 2 Ottawa Health Research Institute, Ottawa, Ontario, Canada; 3 Ottawa Hospital Regional Cancer Centre, Ottawa, Ontario, Canada

**Keywords:** thymidylate synthase, CDK4, p16INK4A, E2F, G_1_ phase

## Abstract

Thymidylate synthase (TS) is the enzyme that catalyses the last step in *de novo* thymidylate synthesis. It is of interest clinically because it is an effective target for drugs such as 5-fluorouracil, often used in combination therapy. Despite a number of earlier reports indicating that TS is a cell cycle-dependent enzyme, this remains equivocal. Here, we show that in HCT116 cells synchronised by serum starvation, there is a clear dissociation between the expression of cyclin E (a well-characterised cell-cycle protein) and TS. Although both cyclin E and TS mRNA and protein increased during G_1_, TS upregulation was delayed. Moreover, TS levels did not decrease following S-phase completion while cyclin E decreased sharply. Similarly, clear differences were seen between cyclin E and TS as asynchronously growing HCT116 cells were growth-inhibited by low-serum treatment. In contrast to previous reports using rodent cells, adenovirus-mediated over-expression of E2F1 and cyclin E in three human cell lines had no effect on TS. Cell-cycle progression was blocked by treatment of cells with pharmacological inhibitors of CDK2 and CDK4 and by ectopic expression of p16INK4A. Whereas CDK2 inhibition had no effect on TS levels, inhibition of CDK4 was associated with decreased TS protein levels. These results provide the first evidence that drugs targeting CDK4 may be useful with anti-TS drugs as combination therapy for cancer.

For DNA synthesis to proceed normally, a balanced pool of the four deoxyribonucleotides, dCMP, dGMP, dAMP and dTMP is required. These deoxyribonucleotides are synthesised from ribonucleotides by the enzyme ribonucleotide reductase (RNR). Of the four deoxyribonucleotides, only dUMP requires further metabolism. It is converted to dTMP by thymidylate synthase (TS), which catalyses the transfer of a methyl group from the co-factor tetrahydrofolate to the C5 position of the uracil moiety. TS is the sole cellular enzyme capable of *de novo* synthesis of dTMP. As such, this enzyme has been used for many decades as a target for cancer chemotherapeutic agents. TS protein levels are elevated in some cancers (Haqqani *et al*, 1999; Mizutani *et al*, 2001) and high levels are associated with a poorer response to drugs targeting dTMP synthesis (Johnston *et al*, 1995). Despite extensive studies of TS for many decades, the mechanisms regulating its expression in human cells remain largely undefined.

Since deoxynucleotides are required for DNA synthesis, it is commonly believed that TS is an S-phase-dependent enzyme (Schwartz and Shah, 2005). In mouse fibroblasts synchronised by mitotic selection, TS behaved as a cell cycle-dependent enzyme with mRNA levels undergoing a 5- to 10-fold increase as cells progress from G_1_- to S-phase (Nagarajan and Johnson, 1989). Similarly, in human diploid fibroblasts synchronised by serum starvation, TS mRNA also increased 14-fold at 24 h after cells resumed growth (Ayusawa *et al*, 1986). However, this pattern of regulation is not universally seen (Dolnick, 2000). One report using sorted, asynchronously growing human tumour cell lines suggested that the level of TS protein did not strongly correlate with cell-cycle phases but rather with the proliferative state of the cells (Pestalozzi *et al*, 1995). In murine cells, synchronised in S-phase by aphidicolin treatment, TS mRNA levels remained rather constant throughout most of the cell cycle following drug removal (Matherly *et al*, 1989).

The majority of normal cells in an adult are in a non-dividing state. In response to appropriate stimuli, some of these cells will begin to proliferate. This transition from G_0_ to G_1_ constitutes the point of entry from the ‘resting’ state to the proliferative state. The decision of a cell to enter the cell cycle is made at a time in G_1_ termed the ‘Restriction point’ (Pardee, 1974). Traversing this checkpoint constitutes an irreversible commitment to cell-cycle progression. Quiescent cells can be stimulated to proliferate by mitogenic signals that activate cyclin D-CDK4/6 complexes. As G_1_ progresses, pRb is phosphorylated by these complexes (Weinberg, 1995; Blagosklonny and Pardee, 2002), ultimately leading to E2F release. E2F transcription factors play an essential role in cell-cycle progression and are directly involved in the regulation of genes required for the G_1_/S transition as well as genes related to DNA synthesis (DeGregori *et al*, 1995). E2F can bind DNA as a homodimers or heterodimer containing the co-activator Dp1 to activate the transcription of target genes such as cyclin E. Newly synthesised cyclin E then forms a complex with CDK2 to drive S-phase entry (Sauer and Lehner, 1995; Woo and Poon, 2003).

Some reports have suggested that E2F is involved in TS regulation. In rodent cells, microarray analysis has shown that ectopic expression of various E2F family members increase TS mRNA levels (Ishida *et al*, 2001; Kalma *et al*, 2001; Polager *et al*, 2002). In human cells, two E2F consensus binding sites are found in the inverted repeat of the TS promoter. However, point mutation of neither of these sites affected TS gene expression (Dong *et al*, 2000; Lee and Johnson, 2000). Although studies of human tumour samples have examined the correlation between E2F levels and TS levels, no consistent picture has emerged. Some studies have shown a strong correlation between expression levels of E2F and TS while others have shown no significant relationship (Kasahara *et al*, 2000; Sowers *et al*, 2003a; Belvedere *et al*, 2004). Regulatory sequences in the TS promoter are highly conserved across species, including mouse and human (Lee and Johnson, 2000). An interesting difference in TS induction between murine and human cells was seen following cytomegalovirus (CMV) infection. Upon CMV infection, TS levels were upregulated in both murine and human cells. However, in mouse cells TS induction was E2F dependent (Gribaudo *et al*, 2000) whereas in human cells it was not (Gribaudo *et al*, 2002). Given the inconsistent pattern of TS regulation in various cell types and the critical role of this enzyme in cancer treatment, we undertook to investigate the relationship between TS expression and the cell cycle in human tumour cell lines.

In the present report, we describe the use of four human cell lines, HCT116, MCF-7, PC3 and GM38, to investigate the cell cycle dependency of TS. HCT116 cells were synchronised by serum starvation and the levels of both TS mRNA and protein were studied and compared to the levels of cyclin E as cells were starved or stimulated to re-enter the cell cycle. The regulatory role of E2F and cyclin E in TS expression was directly assessed by adenovirus-mediated over-expression of these two proteins. We also used small molecule inhibitors of CDKs to block the cell cycle and determine their effect on TS expression levels. Our results provide additional evidence that TS levels in human cells are not dependent on cell-cycle progression but rather on cellular events taking place in G_1_ prior to E2F release and cyclin E activation. TS levels were downregulated by inhibition of CDK4 but not CDK2, suggesting that this cyclin-dependent kinase active only in early G_1_, is involved in the control of TS expression. Additionally, inhibition of CDK4 by p16INK4A over-expression also led to a decrease in TS levels. These results suggest that CDK4 might be a useful target to evaluate clinically in combination therapy with anti-TS drugs.

## MATERIALS AND METHODS

### Cell culture, cell synchronisation and flow cytometry

HCT116 colon cancer cells, MCF-7 and PC3 were obtained from ATCC (Manassas, VA, USA). Normal fibroblast GM38 cells were obtained from NIGMS (Bethesda, MD, USA). Cells were regularly tested for mycoplasma contamination by staining with Hoechst 33258. HCT116 were maintained in McCoy's 5A medium (Wisent, St-Bruno, Quebec, Canada) supplemented with 10% fetal calf serum (FCS, Wisent) at 37°C in a 5% CO_2_/95% air atmosphere. MCF-7, PC3 and GM38 cells were maintained in DMEM (HyClone, Logan, UT, USA) supplemented with 10 and 15% FCS, respectively, at 37°C in a 5% CO_2_/95% air atmosphere. For synchronisation experiments, HCT116 cells were plated at low density (1 × 10^6^ per 10 cm plate) and incubated for 7 days in culture medium containing 0.5% FCS. Following growth arrest, cells were stimulated to re-enter the cell cycle by addition of fresh medium supplemented with 20% FCS. For flow cytometry analysis, cells were harvested by trypsinisation, washed once with cold phosphate-buffered saline (PBS) and fixed by suspending the cell pellet in cold 70% ethanol for 1 h or longer. Cells were then washed with cold PBS and incubated with propidium iodide (1 *μ*g ml^−1^) in PBS containing 40 *μ*g ml^−1^ ribonuclease A for 1 h on ice. Stained cells were then analysed using a BD LSR flow cytometer, appropriately gated to isolate the single cell population. Cell-cycle distribution was then determined using the Modfit program (Verity Software Inc., Topsham, ME, USA).

### Drug treatments and gene transfer

CDK2 Inhibitor III and CDK4 inhibitor have been previously described (Bhattacharjee *et al*, 2001; Zhu *et al*, 2003). Roscovitine, CDK2 Inhibitor III and CDK4 inhibitor were purchased from Calbiochem (Darmstadt, Germany) and dissolved in DMSO. MCF-7, PC3 and HCT116 were plated at a density of 2 × 10^5^ cells per ml incubated overnight prior to addition of the drugs. Adenoviruses encoding cyclin E, Dp1 and E2F1 were supplied by Dr JR Nevins and have been previously described (Schwarz *et al*, 1995; Ekholm *et al*, 2001). CA35 adenovirus encoding *β*-galactosidase was provided by Dr C Addison. Adenovirus expressing human p16INK4A was from Dr F Graham and has been previously described (Schreiber *et al*, 1999). Adenoviruses were propagated in 293 cells and purified by CsCl banding as previously described (Ng and Graham, 2002).

### RNA extraction, RT–PCR and real-time PCR

RNA extractions were carried out as previously described (Birnboim, 1988). cDNA synthesis was carried out using 3 *μ*g total RNA, 1 *μ*g oligo-dT primer (Invitrogen, Carlsbad, CA, USA) and 200 U of M-MLV reverse transcriptase (Invitrogen), following the manufacturer's instructions. Quantification of human TS, Cyclin E and ribosomal protein L32 (RPL32) mRNAs was carried out using the following primer pairs: TS (forward 5′-CCCTGACGACAGAAGAA; reverse, 5′-TAGTTGGATGCGGATTG); cyclin E (forward 5′-CAAGTACACCAGCCACCTC; reverse 5′-GTACAACGGAGCCCAGAA); RPL32 (forward 5′-GCCCTCAGACCCCTTGTG; reverse 5′-AGATGCCAGATGGCAGTTT); E2F1 (forward 5′-GCCACTCGGCTGACGG; reverse 5′-GGCTGATCCCACCTACGGT); *β*-2-microglobulin (forward 5′-CGCTACTCTCTCTTTCTGGC; reverse 5′-AACTTCAATGTCGGATGGAT).

Real-time PCR reactions were performed on a Roche (Basel, Switzerland) Lightcycler in a total volume of 20 *μ*l. The PCR buffer contained 2–4 mM MgCl_2_, 2–8 pmol of each primer, 2 U of Taq polymerase (Invitrogen), 1/40 000 dilution of Sybr green I (Molecular Probes, Eugene, OR, USA), and 2 *μ*l of an appropriate dilution of the cDNA template. Amplification conditions were as follows: for TS, 98°C, 20 s (1 cycle); 98°C, 3 s, 51°C, 8 s, 72°C, 17 s (40 cycles). For cyclin E, 98°C, 20 s (1 cycle); 98°C, 1 s, 60°C, 6 s, 72°C, 24 s (40 cycles). For RPL32, 98°C, 20 s (1 cycle); 98°C, 1 s, 61°C, 6 s, 72°C, 19 s (40 cycles). For E2F1, 99°C, 20 s (1 cycle); 99°C, 3 s, 58°C, 8 s, 72°C, 20 s (40 cycles). For *β*-2-microglobulin, 95°C, 30 s (1 cycle); 95°C, 1 s, 52°C, 10 s, 72°C, 6 s (40 cycles). Relative quantification of unknown samples was achieved by building a standard curve from a two-fold serial dilution series of one of the samples designated as a reference. Values obtained for individual mRNAs were then normalised to data obtained for RPL32 or *β*-2-microglobulin, used as references in these experiments.

### Protein extraction, immunoblotting and densitometry

For western blot analysis, total protein extracts of HCT116, MCF-7, PC3 and GM38 cells were prepared by lysing cells in sodium dodecyl sulphate (SDS) sample buffer (125 mM MOPS, pH 6.8, 2% SDS, 10% glycerol, 1% *β*-mercaptoethanol, 0.001% bromophenol blue) and heating the extracts at 100°C for 10 min.

Following protein quantification using fluorescamine (Udenfriend *et al*, 1972) (Sigma, St Louis, MO, USA), 20–40 *μ*g of total protein extract was resolved on a 12% polyacrylamide gel (19 : 1 acrylamide : bisacrylamide) and transferred on an Immobilon polyvinylidene difluoride membrane (Millipore, Billerica, MA, USA) as previously described (Bissoon-Haqqani *et al*, 2006). Membranes were blocked and then probed with the appropriate dilutions of primary antibodies specific to cyclin E, cyclin D1, E2F1 (Santa Cruz Biotechnologies, Santa Cruz, CA, USA), *β*-actin, p16INK4A (Sigma), p27KIP1 (BD Biosciences, Franklin Lakes, NJ, USA) and rabbit anti-TS (Haqqani *et al*, 1999) (Rockland Immunochemicals, Gilbertsville, PA, USA). Blots were then washed 5 times with TBS-T (10 mM Tris-HCl pH 8, 150 mM NaCl, 0.05% Tween 20) and incubated for 1 h with Envision labelled polymer mouse rabbit-HRP (Dako Cytomation, Glostrup, Denmark) secondary antibody, diluted 1 : 250 in TBS-T.

For densitometry analysis, blots were scanned using a GS 800 densitometer (Bio-Rad, Hercules, CA, USA) and quantification was achieved using the Quantity One software. Bands were quantified and compared to the 0 h signal. *β*-actin levels were used as a reference to correct for loading.

## RESULTS

### Synchronisation of HCT116 cells by serum starvation and re-feeding

**Figure 1:**
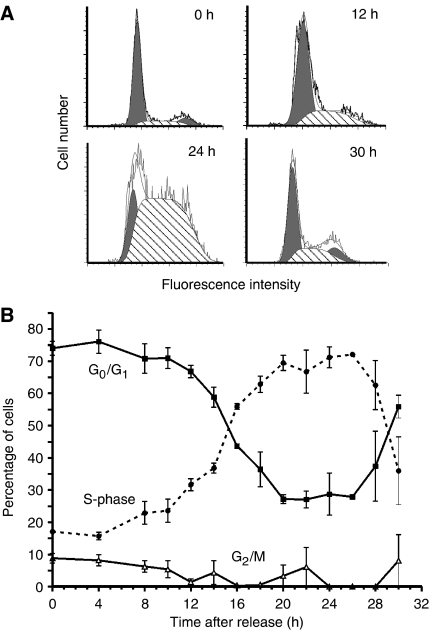
Cell-cycle distribution of synchronised HCT116 following release from serum starvation. (**A**) Histogram analysis of cells stained with propidium iodide at various time points following release. Data were acquired on a BD LSR flow cytometer and analysed using Modfit. The proportion of cells in G_1_ is represented by a black peak on the left and cells in G_2_/M on the right in each panel; cells in S-phase are represented by the hatched area. (**B**) Time-dependent changes in cell-cycle distribution following re-addition of serum. Percentage of cells in each of the cell-cycle phases was determined by Modfit analysis. Data shown are the mean and s.e.m. of three independent experiments.

Synchronisation of HCT116 cells was achieved by maintaining cells for 7 days in 0.5% FCS. Under these conditions, more than 80% of the cells were in the G_0_/G_1_ phase of the cell cycle (Figure 1A). Eight–ten hours after addition of fresh medium supplemented with 20% FCS, cells resumed growth and started to enter S-phase. By 18 h post-stimulation, the majority of cells were actively replicating and, by 30 h, most cells had progressed through the G_2_/M phase and were back in G_1_ (Figure 1A and B). Based on flow cytometry to measure cell-cycle distribution, these conditions achieved an adequate level of cell synchrony. As shown in Figure 1B, the majority of cells entered S-phase between 10 and 16 h post-stimulation, corresponding to the G_1_/S transition. The proportion of cells in S-phase remained high until about 24 h, at which time cells entered G_2_/M. The completion of the first cell cycle and an increase in the G_1_ population was observed at 28 h.

### Kinetics of expression of cyclin E and thymidylate synthase in synchronised HCT116 cells

**Figure 2:**
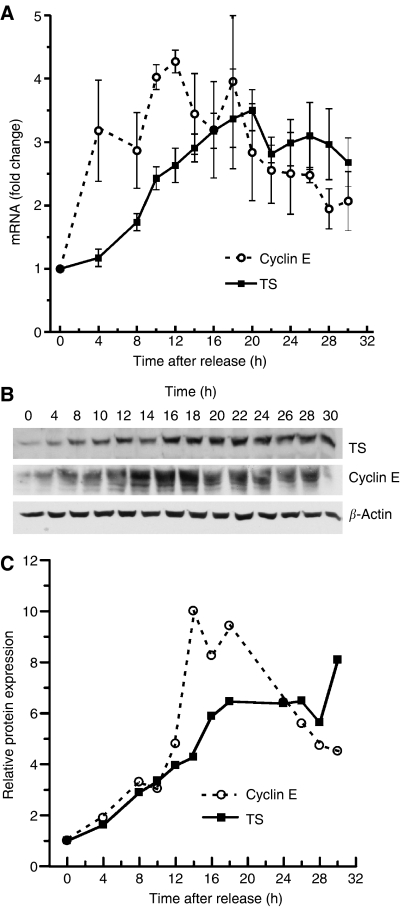
Time course of expression of thymidylate synthase (TS) and cyclin E in synchronised HCT116 cells. (**A**) Time-dependent changes in cyclin E and TS mRNA. Cyclin E and TS mRNA levels were quantified by real-time PCR and normalised to levels of RPL32 mRNA. Data from three independent experiments are shown. Error bars represent the s.e.m. (**B**) Western blot analysis of cyclin E and TS. (**C**) Relative expression of cyclin E and TS protein levels as determined by densitometry of blots shown in (B). Expression levels were normalised to 0 time and to *β*-actin levels.

The expression patterns of human TS and cyclin E mRNA and proteins were compared in HCT116 cells after release from serum starvation. The cyclin E promoter contains a number of E2F consensus sites (Geng *et al*, 1996) and is known to be expressed in late G_1_, at the time of E2F release (Halaban, 1999). In our experimental model, cyclin E mRNA rose sharply by 4 h post-stimulation and reached a maximum (four-fold above baseline) by 10–12 h (Figure 2A). In contrast, changes in TS mRNA followed different kinetics; upregulation of TS mRNA occurred more slowly than cyclin E, reaching its maximum only after 16–20 h. Cyclin E mRNA levels started to drop after 12 h whereas TS mRNA levels remained relatively constant. Cyclin E and TS protein levels, as detected by western blot analysis, also followed different kinetics (Figure 2B and C). The level of both proteins increased in parallel, shortly after re-feeding. Cyclin E increased continuously to a maximum between 14 and 18 h, decreasing thereafter. In contrast, TS increased gradually to a maximum at 16–18 h, remaining relatively constant thereafter. Thus, in this human colorectal cancer cell line synchronised by serum starvation and re-feeding, prominent upregulation of cyclin E and TS was seen in G_1_, reaching a maximum in early S-phase. However, expression of the two genes differed significantly later in the cell cycle: cyclin E mRNA and protein decreased whereas TS did not.

### Kinetics of expression of cyclin E and thymidylate synthase in HCT116 cells during serum deprivation

**Figure 3:**
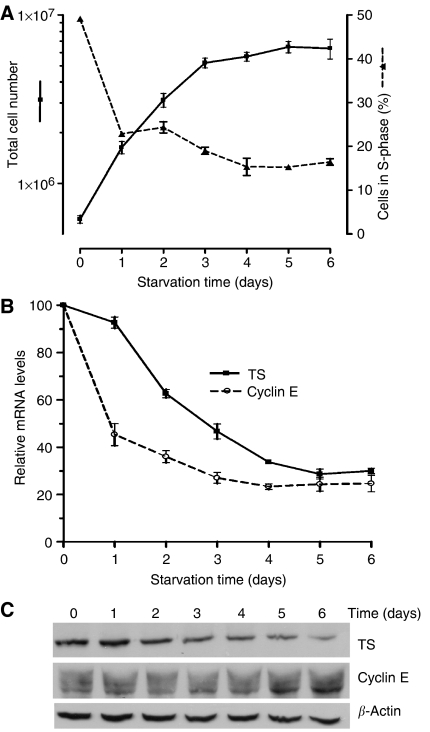
Time-dependent changes in growth rate and expression of thymidylate synthase (TS) and cyclin E in HCT116 cells during starvation. (**A**) Cell number and percentage of cells in S-phase as a function of time. Viable cell number was determined by trypan blue exclusion. The percentage of cells in S-phase was determined by flow cytometry as in Figure 1. The mean and s.e.m. of three independent experiments is shown. (**B**) Time-dependent decrease in TS and cyclin E mRNA in starved HCT116 cells. TS and cyclin E mRNA levels were quantified by real-time PCR and normalised to levels of RPL32 mRNA. The mean and s.e.m. of three independent experiments is shown. (**C**) Western blot analysis showing changes in TS and cyclin E during the course of starvation.

To complement our study of HCT116 cells released from synchronisation by re-addition of serum, we also studied the same cells as they exited the cell cycle following serum deprivation. We followed TS and cyclin E levels periodically during the 6-day course of serum deprivation. During serum deprivation, cells continued to grow rapidly for the first 3 days; thereafter the growth rate decreased appreciably (Figure 3A). The percentage of cells in S-phase, as measured by flow cytometry, decreased sharply at day 1, continued to decrease at a slower rate until day 4, then reached a plateau value of about 15% until day 6 (Figure 3A). The expression of cyclin E and TS was measured during this same period. For TS, both mRNA and protein levels decreased about five-fold almost linearly over the 6-day period (Figure 3B and C). Cyclin E mRNA levels decreased more rapidly than TS mRNA, most evident at days 1 and 2, followed by a more gradual decrease. Changes in cyclin E protein levels did not correlate with changes in mRNA levels; an increase in the level of cyclin E protein was seen after 5 days. Thus, under these experimental conditions, changes in TS and cyclin E differed significantly, providing further evidence of differences in their regulation.

### Ectopic expression of cyclin E and E2F1 does not induce TS expression in HCT116, MCF-7 or GM38 cells

**Figure 4:**
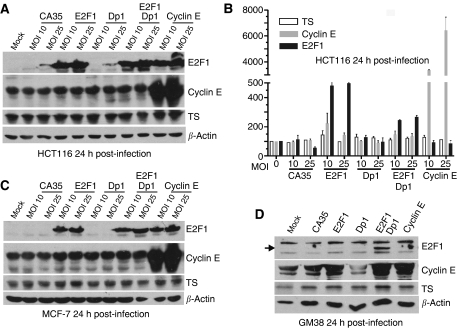
Effect of adenovirus-mediated over-expression of E2F1, Dp1 and cyclin E on thymidylate synthase (TS) expression. (**A**) Western blot analysis of HCT116 cells infected for 24 h at multiplicity of infections (MOIs) of 10 and 25. (**B**) Relative mRNA expression levels of TS, cyclin E and E2F1 24 h after infection with adenoviruses encoding E2F1, Dp1 and cyclin E. mRNA levels were normalised to *β*-2-microglobulin mRNA levels. The mean and s.e.m. of three independent experiments is shown. (**C**) Western blot analysis of MCF-7 cells infected for 24 h at MOIs of 10 and 25. (**D**) Western blot analysis of GM38 cells infected for 24 h at a MOI of 25. Infection with CA35 adenovirus was used as a control in all panels.

Previous reports using mouse and rat embryonic fibroblasts showed that ectopic expression of E2F1 and E2F2 leads to an increase in TS transcripts (DeGregori *et al*, 1995; Ishida *et al*, 2001). To further characterise the relationship between key effectors of G_1_/S transition and the regulation of human TS transcription, we infected HCT116 and MCF-7 cells with recombinant adenoviruses expressing E2F1, Dp1 and cyclin E. Western blot analysis revealed that, 24 h post-infection, both cyclin E and E2F1 were over-expressed in both cell lines (Figure 4A and C). However, no significant changes were observed in TS levels following infection with any of the viruses. Real-time PCR analysis also showed a dose-dependent increase in E2F1 and cyclin E mRNA expression in infected HCT116 cells (Figure 4B). E2F1 mRNA did not accumulate as much as cyclin E mRNA, likely because of the pro-apoptotic activity of E2F1. As expected, ectopic expression of E2F1 alone or E2F1 together with Dp1 (its binding partner), upregulated endogenous cyclin E mRNA by a factor of two—three-fold, but little change was observed in endogenous TS mRNA (Figure 4B). The increase in cyclin E protein levels observed in HCT116 and MCF-7 cell lines infected with E2F1 and E2F1+ Dp1 was less than expected (Figure 4A and C), possibly because these are very fast-growing cell lines. We therefore infected a normal human fibroblast line, GM38, with the same adenoviruses. These cells, which have a longer doubling time, responded to ectopic expression of E2F1 and E2F1+Dp1 by a marked increase in cyclin E levels (Figure 4D). However, despite the observed high E2F1 activity, there was no significant increase in TS protein levels.

### Effects of drug inhibition of CDK4 and CDK2 on cell-cycle progression and TS levels

**Figure 5:**
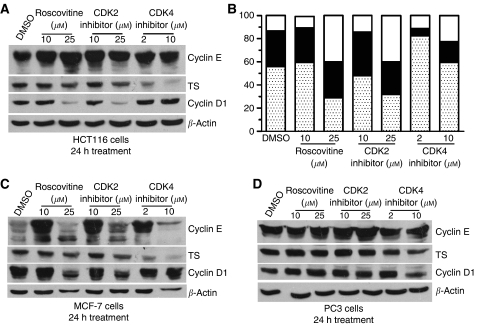
Effect of small molecule inhibitors of cyclin-dependent kinases (CDKs) on HCT116, MCF-7 and PC3 cells. Western blot analysis of thymidylate synthase (TS), cyclin E and cyclin D1 following 24 h treatment with solvent (dimethyl sulphoxide (DMSO)), roscovitine, CDK2 inhibitor or CDK4 inhibitor of HCT116 (**A**), MCF-7 (**C**) and PC3 (**D**) cells. (**B**) Cell-cycle distribution in HCT116 cells following 24 h treatment with the indicated inhibitor. Cell-cycle distribution was determined by flow cytometry as in Figure 1. Percentage of cells in G_0_/G_1_ is represented by the speckled bar, S-phase by the black bar and G_2_/M by the white. Data shown are the average of two independent experiments.

Roscovitine is a potent broad-specificity inhibitor of cyclin-dependent kinases (CDKs), and has been shown to block cell-cycle progression both in G_1_ and G_2_ (Meijer *et al*, 1997). If TS expression were dependent on the cell cycle, then its expression would be expected to be affected by inhibitors of CDKs. The effects of roscovitine and other more specific inhibitors of CDK2 and CDK4 on TS expression were tested on HCT116, MCF-7 and PC3 cells. Following treatment of cells for 24 h with the various agents, expression levels of TS and cell-cycle proteins were determined by western blot analysis and cell-cycle distribution was measured by flow cytometry. As expected, treatment of HCT116 cells with roscovitine increased the G_2_/M fraction and decreased the G_1_ fraction (Figure 5B). Treatment with CDK2 inhibitor yielded very similar results, with an increase in the number of cells in G_2_/M. Treatment with CDK4 inhibitor led to an accumulation of cells in G_0_/G_1_. The changes in cell-cycle distribution in MCF-7 cells treated with the same inhibitors were very similar (data not shown). Inhibition of CDK2 by roscovitine or CDK2 inhibitor was associated with a marked decrease in cyclin D1 protein levels in HCT116 (Figure 5A), a moderate decrease in MCF-7 (Figure 5C), but no change in PC3 (Figure 5D). No consistent pattern of changes in cyclin E expression was seen in the three cell lines in response to treatment with the inhibitors. However, upon treatment with even the lowest dose of CDK4 inhibitor (but not the CDK2 inhibitor), a clear decrease in TS protein levels was observed in all three cell lines. Under the same conditions, no changes in cyclin E or cyclin D levels were detected, except for a decrease in cyclin E in MCF-7 cells treated with 10 *μ*M CDK4 inhibitor. Thus, TS expression in human cells appears not to be related to either cyclin D1 or cyclin E expression levels. Inhibition of CDK4 blocked cell cycle in G_0_/G_1_ and led to a consistent decrease in TS expression in all three cell lines without significantly affecting other cell cycle-dependent proteins. These results strongly suggest that CDK4 activity is involved in the regulation of TS expression.

### Effects of CDK4 inhibition by p16INK4A expression on cell-cycle progression and TS levels

**Figure 6:**
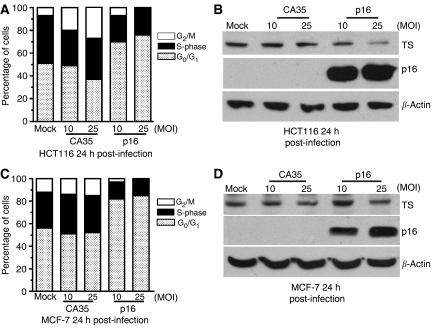
Effect of adenovirus-mediated over-expression of p16INK4A on thymidylate synthase (TS) expression and cell-cycle progression. (**A**) Cell-cycle distribution 24 h after infection of HCT116 cells with adenoviruses encoding Lac Z and p16INK4A at multiplicity of infections (MOIs) of 10 or 25. Cell-cycle distribution was determined by flow cytometry as indicated in Figure 1. Data shown are the average of two independent experiments. (**B**) Western blot analysis of HCT116 cells 24 h after infection with CA35 or p16INK4A adenoviruses at MOIs of 10 or 25. (**C**) Cell-cycle distribution following 24 h after infection of MCF-7 cells with adenoviruses encoding Lac Z and p16 at MOIs of 10 or 25. Cell-cycle distribution was determined by flow cytometry as in Figure 1. Data shown are the average of two independent experiments. (**D**) Western blot analysis of MCF-7 cells 24 h after infection with CA35 or p16INK4A adenoviruses at MOIs of 10 or 25.

To provide additional evidence about the role of CDK4 in the regulation of TS, we infected HCT116 and MCF-7 cells with an adenovirus encoding human p16INK4A, a known inhibitor of CDK4 and CDK6. As expected for an inhibitor of CDK4, HCT116 responded to p16INK4a over-expression by an increase in the G_0_/G_1_ fraction (Figure 6A). Western blot analysis of extracts of infected HCT116 cells showed a strong induction of p16 (Figure 6B). As shown above using a small molecule to inhibit CDK4, over-expression of p16 to inhibit CDK4 also produced a decrease in endogenous TS protein levels. Very similar results were seen in MCF-7 cells (Figure 6C and D). These data demonstrate that inhibition of CDK4 activity either by p16 or by treatment with a small molecule inhibitor leads to an accumulation of cells in G_1_ and produces a decrease in TS. Therefore, CDK4 activity appears to be essential for expression of TS in human cell lines.

## DISCUSSION

The present study was undertaken to address the question of whether TS in human cells is or is not a cell cycle-dependent enzyme. There is a scientific agreement that non-dividing (G_0_) cells have low TS levels, which increase when cells are stimulated to grow. Proliferating cells need balanced levels of the four deoxyribonucleotides for normal DNA replication. The four deoxyribonucleotides are directly synthesised from the corresponding ribonucleotides by the enzyme RNR; however, only dTMP requires an additional step, catalysed by TS, to complete its synthesis. Until recently, RNR was considered to be a prime example of a cell cycle-dependent enzyme. RNR is a dimeric enzyme composed of two non-identical subunits, R1 and R2, encoded by separate genes. R2 is rate limiting for the enzymatic activity of RNR and its level is cell cycle dependent (Chabes and Thelander, 2000). However, an analogue of R2, p53R2 has recently been found that is not cell cycle dependent (Guittet *et al*, 2001; Nordlund and Reichard, 2006). p53R2 can substitute for R2 to form an active RNR enzyme capable of generating deoxyribonucleotides in resting cells or following DNA damage. It is important that RNR and TS have to be coordinated; without adequate levels of TS, dUMP could rise to a high enough level to allow its incorporation into DNA in place of dTMP. This is potentially a mutagenic event (leading to a AT → GC transition) since uracil can be recognised, excised and replaced by cytosine by a base-excision DNA repair pathway designed to recognise spontaneous deamination of cytosine (converting it to uracil) in DNA (Lindahl, 2000). Given the danger to the integrity of the genome should dUMP be incorporated into DNA, one might *a priori* assume the necessity of having adequate levels of TS available whenever deoxynucleotides are synthesised by RNR. Based on recent insight that RNR activity can be independent of S-phase, there is therefore sufficient reason to expect that TS activity should also be independent of the cell cycle.

The widespread assumption that TS is cell cycle dependent enzyme has come from studies that, for the most part, have used rodent models. In synchronised murine cells, TS mRNA and TS activity increased as cells reach S-phase (Navalgund *et al*, 1980; Nagarajan and Johnson, 1989). Ectopic over-expression of E2F transcription factors leads to upregulation of TS transcripts (Ishida *et al*, 2001; Kalma *et al*, 2001; Polager *et al*, 2002). Since E2F transcription factors are one of the main effectors of the G_1_/S transition (Fan and Bertino, 1997) that control the expression of a number of genes required for DNA synthesis (DeGregori *et al*, 1995), these studies reinforced the hypothesis that TS is a S-phase-dependent enzyme. There are, however, other studies which do not support this hypothesis. For example, in asynchronously growing human cancer cells, TS levels were high in cycling cells (largely independent of the phase of the cell cycle) and low in confluent cells (Pestalozzi *et al*, 1995).

The present report provides additional supporting evidence that TS expression in human cells is not closely linked to the cell cycle and also that it is not dependent on E2F activity. When serum-deprived HCT116 cells were stimulated to enter the cell cycle, both TS and cyclin E (a known direct target of E2F transcription factors) started to increase several hours after addition of serum (G_1_ and early S-phase). However, TS and cyclin E differed in that the increase in TS mRNA and TS protein was more gradual than the increase in cyclin E and occurred within a few hours later. Moreover, as cells progressed through the cell cycle, TS mRNA and TS protein levels remained high while cyclin E declined. TS and cyclin E expression was also followed in exponentially growing cells subjected to serum deprivation. Again, the pattern of cyclin E and TS expression showed distinct differences. TS protein and mRNA levels declined almost linearly over a 6-day period whereas cyclin E mRNA decreased sharply in the first day of serum deprivation. To directly assess the role of cellular proteins involved in the G_1_/S transition on TS expression, we over-expressed E2F1, Dp1 and cyclin E in human HCT116 and MCF-7 cancer cell lines as well as in GM38 normal fibroblasts. Ectopic expression of these proteins had no discernible effect on endogenous TS expression in any of the studied cell lines, indicating that neither E2F1 nor cyclin E significantly affect TS expression in human cells. Notably, in normal human fibroblasts, expression of E2F1 and E2F1+Dp1 led to a strong accumulation of endogenous cyclin E, due to increased E2F1 activity, but no change in TS protein expression was observed. Our results, therefore, do not support a role for E2F in TS expression.

We next explored a pharmacological approach to perturbing the cell cycle. Treatment of three cell lines with roscovitine and CDK2 inhibitor led to an accumulation of cells in G_2_/M without an observable effect on endogenous TS levels. However, a marked decrease in TS expression was seen upon treatment with a CDK4 inhibitor. To provide independent evidence that CDK4 activity is indeed linked to TS expression, we carried out experiments using adenovirus-mediated expression of p16INK4A. This cell-cycle inhibitory protein is well known to bind to CDK4 and CDK6 and inhibit their activities (Vidal and Koff, 2000). Over-expression of p16INK4A blocked MCF-7 and HCT116 cells in G_0_/G_1_ and produced a clear decrease in TS levels. Ectopic expression of p16 produced a decrease in TS, confirming the role of CDK4 in the control of TS expression.

**Figure 7:**
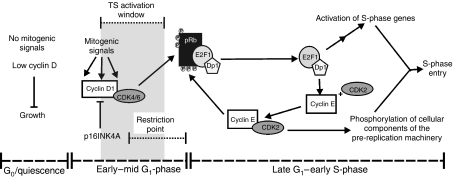
Model of the G_1_ phase of the cell cycle showing the Restriction point and postulated TS activation window. In the absence of mitogenic signals, cells are in a resting (G_0_) state. Upon stimulation with growth factors, cells enter G_1_ and cyclin D levels increase. As G_1_ progresses, cyclin D-CDK4/6 complexes phosphorylate pRb leading to the release of E2F/Dp1 complexes. E2Fs then activate the transcription of a series of S-phase genes and cyclin E. Once cyclin E is synthesised, it associates with CDK2 and drives S-phase entry. The TS activation window is proposed to coincide with the period that CDK4 is active.

The cell cycle is a highly complex process that is only partially understood. Dysregulation of cell-cycle proteins is often found in malignant cells. In cycling cells, E2F1 and cyclin E expression normally reaches a maximum in late G_1_/early S-phase after passing through the Restriction point (Figure 7). Our experiments clearly demonstrate that TS expression in human cells is independent of E2F1 and cyclin E. We show, for the first time, that inhibition of CDK4 leads to a decrease in TS protein. This suggests that there exists a TS activation window in early-mid G_1_ prior to the G_1_/S transition and the activation of CDK2 (Figure 7). Since TS levels are low only in non-dividing cells and elevated in proliferating cells (largely independent of the cell-cycle phases), we propose that TS should be considered a proliferation marker, similar to Ki-67 and PCNA. In the past, the level of expression of E2F1 has been used as a prognostic marker for response to anti-TS drugs on the assumption that TS is an S-phase enzyme (Banerjee *et al*, 2000; Kasahara *et al*, 2000; Sowers *et al*, 2003b). The evidence presented in this report clearly indicates that, in human cells, TS is strictly expressed in early-mid G_1_ and is not regulated by E2F1 activity. Therefore, levels of E2F may not be a valid predictive marker for tumour response to anti-TS drugs (Belvedere *et al*, 2004). One important conclusion from the evidence presented in this and other recent studies employing human cell lines is that there are species differences between human and rodent cells with respect to the regulation of TS. Despite the apparent similarity of regulatory sequences in the TS promoter of the rat, mouse and human genes, it appears that regulation of TS is significantly different in these species.

TS is the target of a number of cancer chemotherapeutic drugs. Over-expression of TS is one mechanism by which tumours may develop resistance to such drugs (Johnston *et al*, 1995). Previous studies have shown that treatment of cancer cells with the CDK inhibitors UCN-01 and flavopiridol led to a downregulation of TS levels and sensitised cells to 5-fluorouracil (Hsueh *et al*, 1998; Abe *et al*, 2000). UCN-01 is a broad-specificity kinase inhibitor, known to inhibit not only CDKs but also PKC and Chk1 (Akinaga *et al*, 1991; Busby *et al*, 2000). Flavopiridol is a flavonoid that exerts a strong inhibitory activity against CDK2, CDK4 and CDK1 (Carlson *et al*, 1996). In the present study, we have shown a marked decreased in TS levels following inhibition of CDK4 but not CDK2. This suggests that the observed decrease in TS expression following treatment with UCN-01 and flavopiridol was due to inhibition of CDK4 but not other CDKs. In recent years, a number of new chemotherapeutics compounds targeting the cell cycle have been developed to inhibit the growth of cancer cells (Collins and Garrett, 2005). PD 0332991, a specific inhibitor of CDK4/6, showed antitumour activity in xenografts (Fry *et al*, 2004). As shown in this report, TS expression is decreased both by small molecule inhibitors of CDK4 and following over-expression of p16INK4A. Interestingly, it has been previously shown that tumour tissue from patients who responded to fluoropyrimidine-based treatment expressed high levels p16INK4A (Kamoshida *et al*, 2004). Since we have shown that CDK4 regulates TS expression, high levels of p16INK4A would be expected to decrease TS levels and to sensitise cells to anti-TS drugs. The data presented in this report provides a rationale for evaluating combination therapy of anti-TS drugs and CDK4 inhibitors, since these two classes of drugs are expected to act synergistically.
